# NEO212, temozolomide conjugated to NEO100, exerts superior therapeutic activity over temozolomide in preclinical chemoradiation models of glioblastoma

**DOI:** 10.1093/noajnl/vdae095

**Published:** 2024-06-11

**Authors:** Radu O Minea, Thu Zan Thein, Zhuoyue Yang, Mihaela Campan, Pamela M Ward, Axel H Schönthal, Thomas C Chen

**Affiliations:** Department of Neurological Surgery, Keck School of Medicine (KSOM), University of Southern California (USC), Los Angeles, California, USA; Norris Comprehensive Cancer Center, KSOM, USC, Los Angeles, California, USA; Department of Neurological Surgery, Keck School of Medicine (KSOM), University of Southern California (USC), Los Angeles, California, USA; Department of Molecular Microbiology and Immunology, KSOM, USC, Los Angeles, California, USA; USC Clinical Laboratories, KSOM, USC, Los Angeles, California, USA; Department of Pathology, KSOM, USC, Los Angeles, California, USA; Department of Molecular Microbiology and Immunology, KSOM, USC, Los Angeles, California, USA; NeOnc Technologies, Inc., Los Angeles, California, USA; Department of Neurological Surgery, Keck School of Medicine (KSOM), University of Southern California (USC), Los Angeles, California, USA; Department of Pathology, KSOM, USC, Los Angeles, California, USA; Norris Comprehensive Cancer Center, KSOM, USC, Los Angeles, California, USA

**Keywords:** chemoradiation, mismatch repair deficiency, O6-guanine-DNA methyltransferase, radiosensitization, temozolomide

## Abstract

**Background:**

The chemotherapeutic standard of care for patients with glioblastoma (GB) is radiation therapy (RT) combined with temozolomide (TMZ). However, during the twenty years since its introduction, this so-called Stupp protocol has revealed major drawbacks, because nearly half of all GBs harbor intrinsic treatment resistance mechanisms. Prime among these are the increased expression of the DNA repair protein O6-guanine-DNA methyltransferase (MGMT) and cellular deficiency in DNA mismatch repair (MMR). Patients with such tumors receive very little, if any, benefit from TMZ. We are developing a novel molecule, NEO212 (TMZ conjugated to NEO100), that harbors the potential to overcome these limitations.

**Methods:**

We used mouse models that were orthotopically implanted with GB cell lines or primary, radioresistant human GB stem cells, representing different treatment resistance mechanisms. Animals received NEO212 (or TMZ for comparison) without or with RT. Overall survival was recorded, and histology studies quantified DNA damage, apoptosis, microvessel density, and impact on bone marrow.

**Results:**

In all tumor models, replacing TMZ with NEO212 in a schedule designed to mimic the Stupp protocol achieved a strikingly superior extension of survival, especially in TMZ-resistant and RT-resistant models. While NEO212 displayed pronounced radiation-sensitizing, DNA-damaging, pro-apoptotic, and anti-angiogenic effects in tumor tissue, it did not cause bone marrow toxicity.

**Conclusions:**

NEO212 is a candidate drug to potentially replace TMZ within the standard Stupp protocol. It has the potential to become the first chemotherapeutic agent to significantly extend overall survival in TMZ-resistant patients when combined with radiation.

**Key Points**

- NEO212 overcomes multiple mechanisms of resistance in animal models of glioma.
- The survival gains with NEO212 do not come at the expense of additional toxicities.
- NEO212 chemoradiation has the potential to extend the survival of glioma patients.

**Importance of the Study**

Glioblastoma (GB) remains very challenging to control in the clinic with 5-year survival rates still in single digits. The intrinsic radioresistance of gliomas and the minimal radiosensitization activity of temozolomide (TMZ) in tumors that express O6-guanine-DNA methyltransferase (MGMT) or are deficient in mismatch repair (MMR) proteins are two main contributors to the inability of Stupp protocol to control the progression of GB. The radiosensitization performance of TMZ is particularly blunted by the poor intratumoral concentrations achieved by this alkylating agent at standard dosages. In this study, we evaluated the radiosensitization activity of NEO212—a novel TMZ derivative with superior tumor bioavailability—in animal models of GB. We show that NEO212 has the potential to overcome multiple mechanisms of TMZ resistance and thus better synergize with radiotherapy. We envision that NEO212 will be particularly beneficial for MGMT promoter unmethylated (MGMT expressing) and MMR-deficient gliomas, which are currently not responding to TMZ treatment.

Glioblastoma (GB) is the most common malignant primary brain tumor. The current standard of care consists of surgery, followed by chemoradiation with temozolomide (TMZ).^1^ This latter component—commonly referred to as the *Stupp protocol*—consists of fractionated radiation therapy (RT) administered concurrently with a daily dose of TMZ over 6 weeks, which is followed by monthly cycles of adjuvant TMZ in the absence of further RT. While the therapeutic benefits of this protocol have been well established over the years,^2,3^ a number of limitations have emerged as well.

As was initially observed in the landmark EORTC-NCIC trial, the average survival benefit of adding TMZ to RT was a mere 2.5 months over RT alone; ie, the inclusion of TMZ extended median overall survival (mOS) from 12.1 months (RT alone) to 14.6 months.^1^ However, further analysis revealed that this seemingly small treatment response was greatly influenced by the expression level of O6-methylguanine-DNA methyltransferase (MGMT) protein in the patient’s tumor tissues.^4^ MGMT is a DNA repair protein that is able to remove the toxic O6-methylguanine (O6-mG) lesions that are imposed onto DNA through the alkylating function of TMZ. As a consequence, the cytotoxic impact of TMZ can be neutralized quite effectively when MGMT is present.^5,6^ It was therefore not entirely surprising to find that those patients with epigenetic MGMT gene silencing in their tumor tissues received a substantially greater benefit (mOS = 21.7 months) in response to treatment with the Stupp protocol than patients with an active (unmethylated) MGMT promoter (mOS = 12.7 months). These comparisons were expanded to yield the critical finding that the latter 12.7 months in response to combination therapy were not statistically different from the 11.8 months observed in the group receiving only RT,^4,7,8^ raising the first concerns that TMZ does not appear to unfold significant benefit in patients with active MGMT expression.^9,10^

There are further limitations to using TMZ for the treatment of GB. Beyond MGMT, it has been recognized that a deficiency in DNA mismatch repair (MMR) provides profound TMZ resistance.^11,12^ Somewhat counter-intuitive, a cell’s competence to repair DNA mismatches is critical for the generation of DNA strand breaks in response to the O6-mG lesions set by TMZ. In MMR-proficient cells, the TMZ-generated O6-mG is mispaired with thymine instead of the usual cytosine, which triggers repair attempts by the MMR system. However, MMR is unable to resolve this type of mismatch; eventually, its continuing, but futile attempts cause apoptosis. In contrast, in the absence of MMR, the cell is able to tolerate these mismatches by introducing mutations into the DNA strand.^5,13^ In the context of clinical practice, it has not been well established whether a significant fraction of newly diagnosed GB patients present with MMR deficiency in their tumors. However, a small number of studies provided evidence that recurrent GB more frequently shows MMR deficiency, which could explain why these patients stopped responding to treatment.^14,15^

Another limitation of TMZ-based therapy is this drug’s sub-optimal penetration of the blood-brain barrier (BBB). Although TMZ is considered to be brain penetrant, its brain-blood ratio is only about 0.2,^16^ meaning that the majority of TMZ present in the systemic circulation does not enter the brain parenchyma.^17,18^ This less-than-desirable brain distribution could be among the key aspects contributing to the above-described drawbacks, such as its lack of overcoming MGMT or MMR-based resistance, and absence of clear radiosensitizing potential.^19^ Regrettably, endeavoring to ameliorate these weaknesses through increased dosing of TMZ would not be possible, due to the drug’s dose-limiting myelosuppressive toxicity.^20–22^

In view of the above, one could conjecture that the treatment success of the Stupp protocol could be much improved if its radiation component was combined with a drug that can achieve a high brain-blood ratio, overcome MGMT and MMR-based resistance, and at the same time was well tolerated without severe myelosuppression. With these goals in mind, we are developing a novel chemical compound, NEO212 (NeOnc Technologies, Inc.), that has shown promise to fill this urgent medical need. NEO212 emerged from our in silico-based search for TMZ derivatives with predicted increased BBB penetration ability,^23^ where subsequent experiments established that its brain:plasma ratio is about 3-fold higher than that of TMZ.^24^

NEO212 is the first new chemical entity derived from a bioconjugate platform that conjugates suitable agents to proprietary NEO100 (NeOnc Technologies, Inc.), which is a highly pure version of perillyl alcohol synthesized under current good manufacturing practices (cGMP).^25^ Extensive preclinical studies have characterized NEO212’s remarkable anticancer activity, while at the same time establishing its low toxicity.^26–30^ In the current study, we investigated the activity of NEO212 in combination with RT in different mouse GB models representing resistance mechanisms that may be encountered in GB patients. We attempted to mimic the concurrent phase of the Stupp protocol, and we performed all experiments alongside TMZ as the standard of care.

## Materials and Methods

### Reagents

NEO212 was obtained from Axon Medchem (Groningen, The Netherlands). TMZ was purchased from TCI America (Portland, OR). All other reagents were purchased from Millipore Sigma (Burlington, MA). All antibodies used in the study are listed in Supplementary File.

### Cells

The LN229, T98G, and U251 human GB lines were obtained from ATCC (Manassas, VA). The LN229TR2 is a TMZ-resistant, MMR-deficient variant of LN229 generated after exposing parent cells to increasing concentrations of TMZ.^27^ The TMZ-resistant variant of U251 cells (ie,. U251M) was generated by infecting the parent cells with a lentiviral construct that expresses human MGMT and 2 reporter genes (firefly luciferase (Luc) and enhanced green fluorescent protein). The primary glioma stem cells USC02 (mesenchymal phenotype) and USC04 (pro-neural phenotype) were originally isolated and characterized by our group.^28^ The preparation of USC02 MGMT knockdown cells is described in the supplementary file.

### Analysis of MGMT and MMR Status

MGMT status was investigated by Western blot as described previously.^31^ MMR status was analyzed by 2 approaches: (1) Western blot analysis of MSH2 and MSH6, which are proteins essential for the execution of MMR, and (2) microsatellite instability (MSI) analysis with a DNA amplification kit for multiallelic microsatellite regions. The kit was purchased from Promega (Madison, WI) and was able to screen 8 alleles in total.

### Efficacy Studies in Tumor-Bearing Animals

All studies were conducted under animal protocol #21163, which was approved by the Institutional Animal Care and Use Committee of the University of Southern California. Tumor xenografts were established by implanting GB cells into the right hemisphere (coordinates: A/P–1.00 mm, M/L + 1.00 mm, and D/V–2.50 mm) of athymic mice on a stereotaxic frame (Kopf Instruments, Tujunga, CA). Tumors were allowed to grow for 14 days before treatments were initiated. When applicable, the tumor growth was monitored on an IVIS Spectrum optical imaging system (Perkin Elmer, Shelton, CT). In all animal models, the treatments were administered for 10 days in total (5-days on/2-days off/5-days on). NEO212 and TMZ were dosed at 25 mg/kg/day by oral gavage after being first dissolved in DMSO and then mixed in an OraPlus suspending vehicle. Whole brain RT (WBRT) was administered in an X-RAD320 irradiator (Precision X-Ray, North Branford, CT) and dosed at 2 Gy/day using the following irradiator settings: 250 kV, 16.0 mA, source-to-surface distance 50 cm, and F1 filter (2 mm Al). A radiosensitization factor (RF) was calculated for each treatment modality based on median survival data for each group and using the following formula: survival gains from combination therapy (days)/survival gains from monotherapy 1 (days) + survival gains from monotherapy 2 (days). A ratio of < 1 was considered antagonistic, equal to 1 additive, and > 1 synergistic.

### Histological Analyses of Tissues

Brain and bone marrow slides were prepared from animals with USC02 xenografts and stained with a γH2AX antibody (detecting DNA damage), CD31 antibody (detecting endothelial cells) or were subjected to TUNEL (detecting apoptosis). Bone marrow slides were also stained with a CD45 antibody to visualize the effects of the treatments on the lympho-myeloid cellular compartment. The methods for preparing and analyzing the brain and bone marrow slides are described in the supplementary file.

### Statistical Analysis

Statistical significance was analyzed in Prism v.10.2.1 (GraphPad Software) and assessed by one-way analysis of variance (ANOVA) with a significant overall F-test followed by Tukey or Bonferroni post-hoc multiple comparison tests of treatment groups relative to control. Kaplan–Meier survival curves were also generated in Prism and the log-rank (Mantel-Cox) test was used for comparisons between survival curves. Two-tailed *P* < .05 was considered significant.

## Results

### GB Cells Harbor Different Treatment Resistance Mechanisms

**Figure 1.**
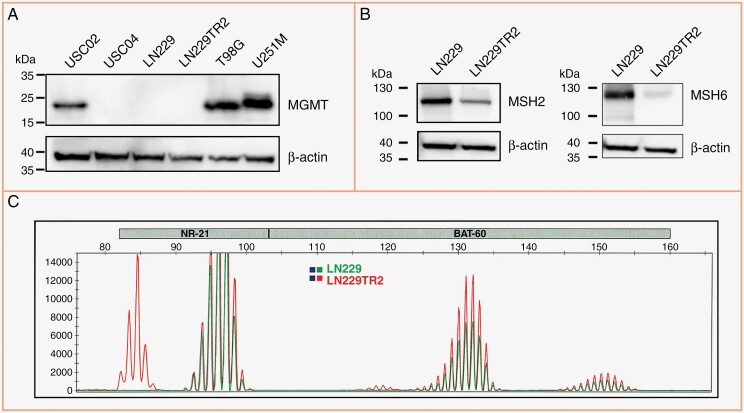
The GB cell models used in this study display different mechanisms of resistance to TMZ. (A) Cell lysates were analyzed by Western blot to determine MGMT protein expression in the various cell models used in this study. T98G cells were included as a positive control and LN229 cells as a negative control; actin was used as the loading control. (B) MSH2 and MSH6 are proteins necessary for MMR function. Western blot comparison of parental LN229 and derived LN229TR2 cells reveals very low MSH2 levels, and near absence of MSH6 protein, indicating MMR deficiency. (C) Analysis of microsatellite instability (MSI) shows evidence of instability in LN229TR2 cells, with novel alleles displayed in 2/8 markers (NR-21 and BAT60), which is yet another indication of MMR deficiency.

For this study, we used 4 GB cell lines harboring different mechanisms of TMZ resistance and varying degrees of radiosensitivity, as follows. USC02 and USC04 represent primary patient-derived GB stem cells that differ in their MGMT protein expression levels (**Figure 1A**) and in their sensitivity to ionizing radiation (see below). U251M cells are derived from the widely used U251 cell line by infection with a lentivirus construct harboring MGMT cDNA, and consequently, these cells express high levels of exogenous MGMT (**Figure 1A**). LN229TR2 cells were selected from their parental LN229 cell line through long-term treatment with TMZ; these cells have lost their MMR function, as indicated by low expression of MMR proteins MSH2 and MSH6 (**Figure 1B**), along with the emergence of microsatellite instability (**Figure 1C**), a characteristic marker of MMR deficiency. The in vitro sensitivity of these 4 cell lines towards TMZ has been characterized in our prior studies and shown to closely align with expectations; ie, MGMT-expressing cells (USC02 and U251M) and MMR-deficient cells (LN229TR2) are robustly TMZ-resistant, whereas USC04 cells are TMZ sensitive.^28,31^

### NEO212 Outperforms TMZ in Treatment Success of Preclinical GB Models

In a prior in vitro study, we had obtained evidence that NEO212 might act as a radiosensitizer, and that this function was significantly stronger than that of TMZ.^31^ In view of the relevance of this issue with regard to the current standard of care for GB patients, we designed in vivo experiments mimicking the concurrent phase of the Stupp protocol. The above-described GB cell types were orthotopically implanted into mice, and tumor development was monitored via bioluminescent imaging (Supplementary Figures S1, S2, and S3). After tumor take had been confirmed in each animal, the mice were subjected to daily treatment with 25 mg/kg NEO212 or 25 mg/kg TMZ, with or without concurrent WBRT over 10 days. Treatment efficacy was monitored through imaging at regular intervals, along with recording of the survival of each animal.

**Figure 2.**
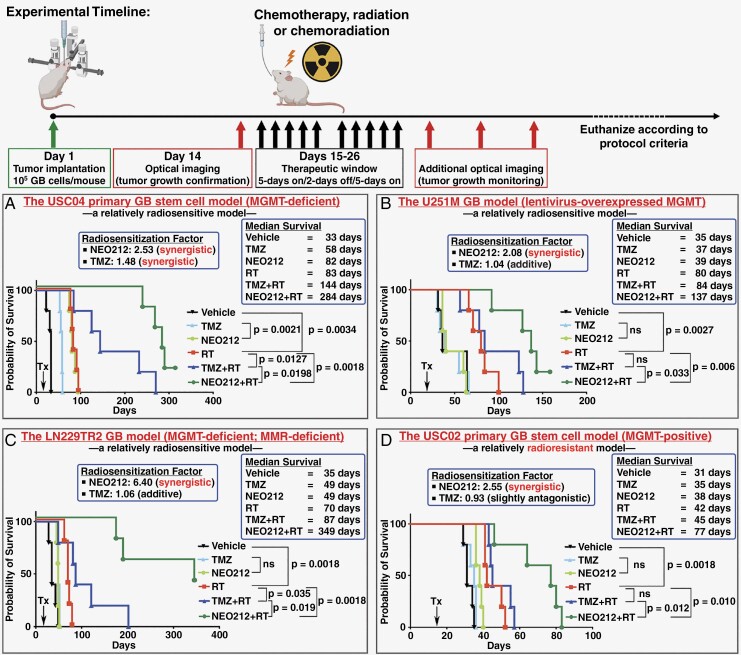
NEO212 synergizes with radiation therapy and prolongs survival of multiple GB models. Kaplan–Meier survival plots were generated to display animal survival in response to various treatments. Groups of 5 mice were treated with NEO212 (25 mg/kg/day) or TMZ (25 mg/kg/day) alone or in combination with radiation therapy (2 Gy/day). Control groups of mice received vehicle only. (A) The USC04 primary GB stem cell model represents the “baseline” model, as it lacks obvious treatment resistance mechanisms. (B) The U251M model expresses high levels of exogenous MGMT and therefore represents a TMZ-resistant model. (C) The LN229TR model represents a model where TMZ resistance is based on MMR deficiency. (D) The USC02 primary GB stem cell model represents a strongly treatment-resistant model, based on expression of endogenous MGMT, along with relative radioresistance. The log-rank (Mantel-Cox) test was used for statistical comparisons. ns: not significant. Some graphical elements used for this figure were imported from BioRender.com.

The treatment outcomes and overall animal survival are summarized in **Figure 2**. The USC04 stem cell model represented the “baseline” model, due to its lack of obvious treatment resistance mechanisms. As presented in **Figure 2A**, this model was responsive to all treatment modalities. When applied in a monotherapy fashion, both NEO212 and TMZ clearly extended survival, although NEO212 had a significantly greater beneficial effect. In combination with RT, both drugs revealed radiosensitizing features and further prolonged survival, although, once again, the beneficial impact of NEO212 was far greater than that of TMZ. The median survival of the group of mice treated with NEO212 + RT was nearly 2 times longer (284 days) than that of mice treated with TMZ + RT (144 days). In essence, while this tumor model responded to TMZ and radiation in a manner that would be expected from a treatment-sensitive patient successfully subjected to the Stupp protocol, NEO212 in comparison still significantly (*P* = .0198) outperformed TMZ in this setting.

We next applied the chemoradiation protocol to the U251M (MGMT overexpressing) model. As shown in **Figure 2B**, this model did not respond to either drug in monotherapy fashion. In combination with RT, TMZ slightly extended median survival to 84 days, as compared to 80 days achieved by RT alone, although this effect was not statistically significant. In comparison, NEO212 was able to extend median survival to 137 days, which was 57 days longer than what was achieved with RT alone (*P* = .006). This benefit of NEO212 was more impressive because NEO212 alone had no significant effect; rather, it reflected its potent radiosensitizing activity (which did not become apparent from TMZ in this model).

Another clinically relevant model, illustrating the role of mismatch repair, was represented by MMR-deficient LN229TR2 cells. Treatment of mice harboring such tumors with TMZ or NEO212 showed a small benefit and extended median overall survival from 35 to 49 days in both cases (**Figure 2C**). On the other hand, the striking radiosensitizing potential of NEO212 once again emerged in the RT combination setting. While TMZ had a small additive effect and extended median survival to 87 days as compared to the 70 days observed with RT alone, NEO212 + RT achieved a median survival of 349 days (*P* = .0018 compared to RT alone). Calculation of NEO212’s RF yielded a very strongly synergistic 6.40, yet again emphasizing this agent’s striking radiosensitization potential.

Finally, we applied our in vivo Stupp protocol to USC02 cells, which are MGMT-expressing GB stem cells, and as such potentially the most difficult to eradicate by therapy. Results in **Figure 2D** show that these cells also harbor inherent radioresistance: RT in this model extended median survival by a mere 11 days over untreated controls. In comparison, the other 3 models used in our study showed an RT benefit from 35 to 40 days over untreated controls (**Figure 2A–C**). Treatment with TMZ or NEO212 as monotherapy modestly extended the survival of the USC02 model by a few days, although for TMZ this effect was not statistically significant. In combination with RT, TMZ did not add any survival benefit, which was consistent with expectations derived from the clinical experience with this tumor phenotype. In contrast, NEO212 + RT once again demonstrated its synergistic potency and significantly extended survival by 35 days over RT alone (*P* = .01). NEO212’s RF was calculated at a strongly synergistic 2.55, further validating this agent’s exquisite sensitizing potential.

The above-described survival gains were consistent with the bioluminescent imaging of tumor growth that was performed at regular intervals (**Supplementary Figures S1, S2, and****S3**). This monitoring by imaging also confirmed that the cause of death of individual mice was cancer-related, rather than due to extraneous circumstances, and it further demonstrated that treatment with NEO212 + RT suppressed the bioluminescent signal—which is indicative of the presence of tumor tissue—far longer than the other treatment regimens.

An additional effort was aimed at understanding whether the presence of MGMT possibly contributed to the relative radioresistance of the USC02 model. This question arose based on an observation in the original EORTC-NCIC trial, noting that overall survival of GB patients with MGMT-overexpressing tumors was shorter in response to RT alone (11.8 months) than survival of RT-only treated patients with tumors not expressing MGMT (15.3 months).^4^ To investigate this issue, we established USC02 cells harboring effectively silenced MGMT (**Supplementary Figure S4**) and subjected them to the same chemoradiation protocol as above. However, the median survival of the different treatment groups was not substantially different (**Supplementary Figure S5**) from that observed in the parental, MGMT-expressing USC02 cells shown in **Figure 2D**. Although this result further validated the superior radiosensitizing potency of NEO212 over TMZ, it did not establish a connection between MGMT status and radiosensitivity in this model.

### Chemoradiation With NEO212 Causes Extensive DNA Damage and Apoptosis in Tumor Cells

**Figure 3.**
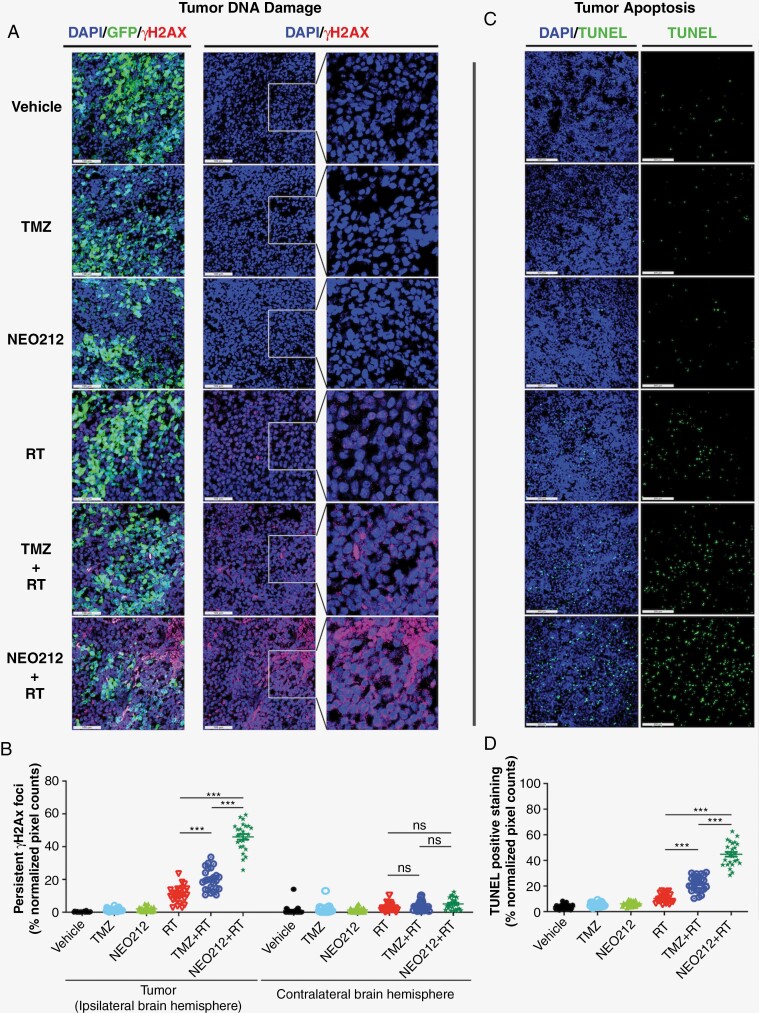
Chemoradiation with NEO212 causes profound DNA damage and apoptosis in tumor tissue but spares normal brain tissue. Mice harboring USC02 tumors were subjected to the same treatment regimen as described in the legend in Figure 2. At the end of the 10-day treatment period, brains were collected and separated into the ipsilateral hemisphere (containing the tumor) and contralateral (tumor-free) hemisphere. Brains were sectioned and analyzed. (A) Sections were stained with AF647-labeled γH2AX antibody to detect DNA damage, and counterstained with DAPI to visualize cell nuclei. Green fluorescence identifies tumor cells, which express a green fluorescent protein (GFP). Scale bars are 100 μm. (B) The graph presents the quantification of DNA damage staining in tumor tissue and tumor-free normal brain tissue. This was done in 30 noncontiguous fields per treatment group with each data point representing the average for one field. (C) Sections were subjected to TUNEL staining to reveal apoptotic cells, and counterstained with DAPI. Scale bars are 200 µm. (D) Quantitative representation of apoptosis in tumor tissue. ns: not significant; 3 asterisks (***): *P* < .001.

The DNA-damaging and apoptosis-inducing effects of all treatments were assessed on brain sections from animals harboring USC02 tumors. To investigate DNA damage, we stained with a fluorescently labeled antibody recognizing γH2AX protein, whose persistent focal presence is a marker for DNA double-strand breaks (DSBs). Representative brain tumor sections are presented in **Figure 3A**, and averages from all sections are quantitatively summarized in **Figure 3B**. These data show an increase in persistent DSBs after RT, and this effect was exacerbated by the inclusion of TMZ or NEO212. Consistent with the survival outcomes of these treatment regimens, chemoradiation with NEO212 caused by far the most DNA damage in the tumor tissue. However, when tissue sections from the contralateral normal brain were analyzed, DNA damage caused by chemoradiation with NEO212 was not elevated as compared to the minor effect that was seen with RT alone (**Figure 3B**), demonstrating that the addition of NEO212 to RT does not trigger increased neurotoxicity.

The extent of apoptosis within tumor tissue was investigated with the standard TUNEL assay. Here as well, RT caused an increase in tumor cell apoptosis, and this effect was exacerbated by inclusion of TMZ or NEO212, with the latter clearly showing the most extensive chemoradiation-induced cell death (**Figure 3C, D**). This once again superior contribution of NEO212 mirrored its DNA-damaging activity and suggested that tumor cell death resulted from the extensive DNA damage caused by chemoradiation with NEO212.

### Chemoradiation With NEO212 Exerts Anti-angiogenic Effects

**Figure 4.**
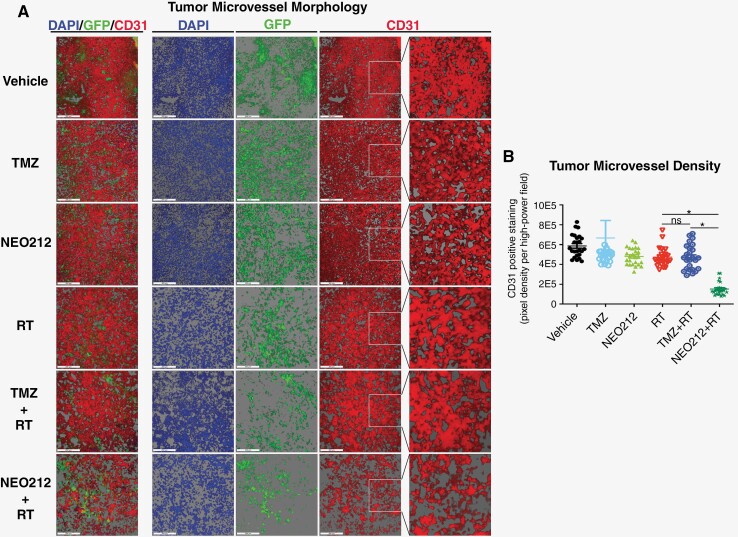
Chemoradiation with NEO212 causes anti-angiogenic effects. Mice harboring USC02 tumors were subjected to the same treatment regimen as described in the legend in Figure 2. At the end of the 10-day treatment period, brain tumors were collected, and sections were analyzed for microvessel density (MVD). (A) Sections were stained with AF647-conjugated CD31 antibody to visualize endothelial cells and z-stacks are shown to illustrate the 3-D microvessel structures. DAPI was used as the counterstain, and the presence of green fluorescent protein (GFP) was used to identify tumor cells. Scale bars are 200 µM. (B) Chart showing the quantitative analysis of CD31 staining. This was done in 30 noncontiguous z-stacks per treatment group with each data point representing the average for one z-stack. ns: not significant; one asterisk (*): *P* < .05.

Extensive neo-vascularization of GB is a characteristic of this tumor type and supports its aggressive growth. We therefore investigated whether our different treatments had an effect on this process by analyzing the microvessel density (MVD) of USC02 tumor sections. We used a fluorescently labeled antibody recognizing CD31, a cell surface marker of endothelial cells that plays a role in neo-angiogenesis. Representative images from tissue staining are shown in **Figure 4A** and quantitative analysis is summarized in **Figure 4B**. These data reveal pronounced ectasia in the treatment groups that received RT, consistent with what is known about the effects of RT on blood vessels.^32^ The addition of TMZ to RT does not appear to contribute an additional effect, whereas the addition of NEO212 to RT dramatically changes the morphology of the tumor vasculature further, showing a pronounced loss of endothelial cells that is not observed under any other treatment conditions. In all, tumor tissue from animals receiving NEO212 chemoradiation presents with a strikingly lower MVD, along with more disorganized and likely nonfunctional blood vessels. While we did not investigate whether this dramatic effect resulted from inhibition of endothelial cell proliferation or tubule formation, the generally increased apoptotic index within the tumor tissue (**Figure 3C, D**) suggests that the killing of tumor endothelial cells could play a role. Of note, as chemoradiation with TMZ does not result in this dramatic morphological change, the effects of chemoradiation with NEO212 not only represent a quantitative difference to TMZ, but a qualitative distinction as well.

### Chemoradiation With NEO212 Does Not Cause Bone Marrow Toxicity

**Figure 5.**
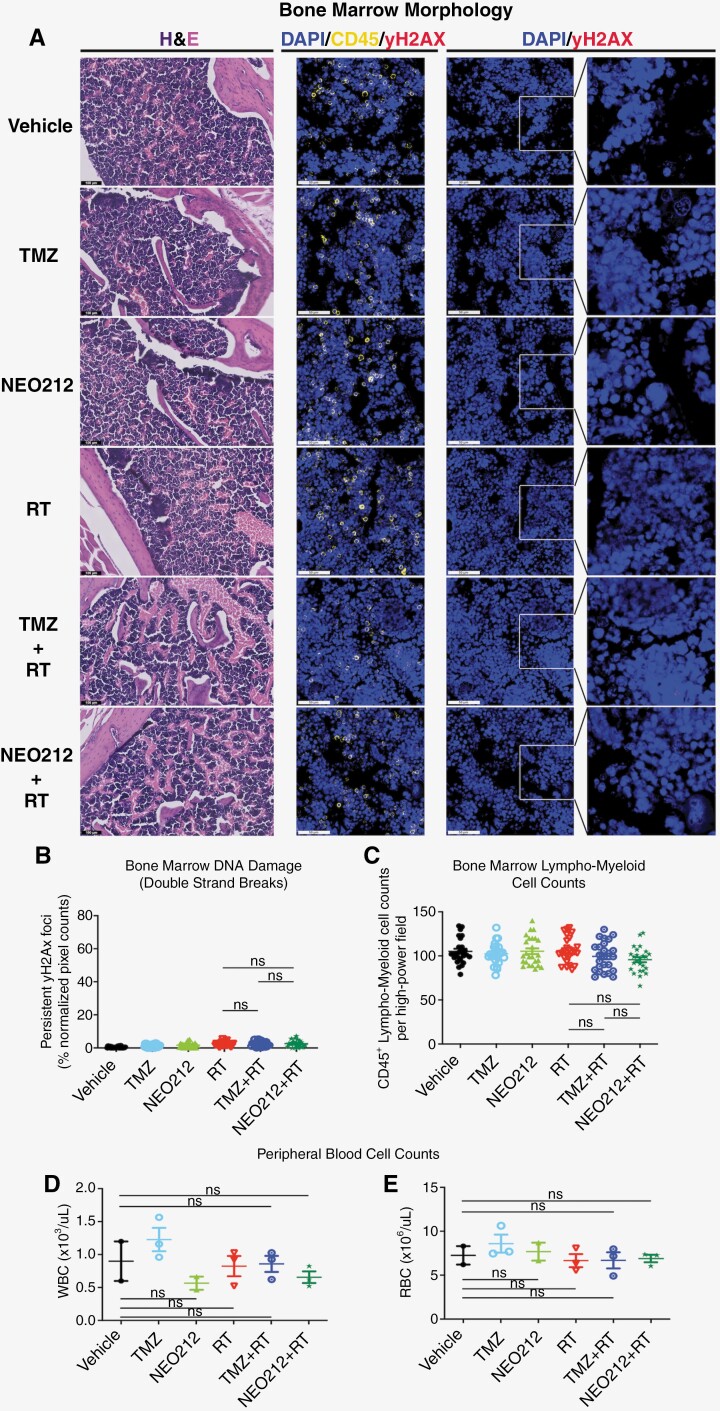
Chemoradiation with NEO212 does not cause bone marrow toxicity. Mice harboring USC02 tumors were subjected to the same treatment regimen as described in the legend in Figure 2. At the end of the 10-day treatment period, bone marrow and blood were collected and analyzed for signs of toxicity. (A) Bone marrow was subjected to H&E staining (to show general cellularity), along with immunohistological analysis with AF647-conjugated γH2AX antibody (to reveal DNA damage) and AF555-conjugated CD45 antibody (to identify hematopoietic cells). DAPI was used as the counterstain. Scale bars are 50 µm for the immunostains and 100 µm for the H&E stains. (B) Quantitative analysis of γH2AX staining. (C) Quantitative analysis of CD45^+^ cell counts. (D) Number of white blood cells (WBC) in the peripheral blood. (E) Number of red blood cells (RBC). ns: not significant.

Bone marrow toxicity represents the main concerning side effect that can arise in GB patients treated with TMZ-based chemoradiation.^20–22^ We therefore investigated this issue in our mouse models. Bone marrow sections prepared from USC02 tumor-bearing mice and exposed to the same treatment sequences as used in the above survival estimations were assessed for DNA damage (γH2AX staining) and for their morphology and cellularity. In addition, peripheral blood cell counts were performed for white and red blood cells (WBC and RBC). Representative bone marrow sections are shown in **Figure 5A**, and related qualitative data are summarized in **Figure 5B–E**. Combined, these data show that none of the treatment conditions revealed a significant toxic impact on the bone marrow. Thus, while chemoradiation with NEO212 was shown to exert strikingly effective therapeutic activity that was significantly greater than chemoradiation with TMZ, NEO212’s superiority was not achieved at the cost of greater toxicity.

## Discussion

Chemoradiation therapy, where RT is combined with TMZ, represents the standard of care for newly diagnosed GB patients. While GB is not considered to be inherently resistant to RT, cancer stem cells often are more resistant than the bulk of the tumor cells and more prone to withstand and survive therapeutic intervention.^33,34^ It therefore has been investigated whether TMZ might be able to contribute any highly desirable radiosensitizing properties to the Stupp protocol. However, experimental analysis of this aspect generated mixed results,^35–38^ and TMZ is not generally accepted as a potent radiosensitizer (see detailed discussion in ref.^19,31^).

Results from our preclinical study provide support for the view that the novel compound NEO212 might hold the potential to significantly improve the success of chemoradiation therapy of GB patients by overcoming several of the limitations of the Stupp protocol that are determined by inclusion of TMZ. Based on these results, NEO212 displays at least 2 key advantages over TMZ: (i) it is active against tumor cells harboring common TMZ-resistance mechanisms, and (ii) it is able to strongly sensitize tumor cells (including cancer stem cells) to radiation. Gratifyingly, the increased activity of NEO212 does not come at the expense of greater toxicity; based on a number of observations, myelosuppression in response to treatment of animals appears no greater than what is observed with TMZ, and other side effects have not become apparent.

For newly diagnosed GB patients, MGMT protein expression in their tumor tissue worsens their prognosis, because they are not likely to receive the full benefit of TMZ in their chemoradiation protocol.^4,7,8^ A large number of preclinical and clinical studies have established MGMT as a highly effective reversal mechanism against the toxic impact of TMZ,^5–7^ and its use as a predictive biomarker has been widely accepted.^39^ In fact, it represents an ongoing discussion whether TMZ should even be given to patients when MGMT protein is present in their tumor tissue, as it might unnecessarily and unproductively increase the risk of side effects.^9,10^ These issues surrounding MGMT were faithfully recapitulated in our mouse tumor models. While our “baseline” USC04 model (tumor cells lacking MGMT) is shown to respond to a single TMZ treatment and also benefits from adding TMZ to RT, the 2 MGMT-overexpressing models demonstrate no benefit. In contrast, NEO212 exerts clear benefit in all models, primarily through its ability to provide pronounced radiosensitizing activity, even when a certain degree of radioresistance is present.

Unlike MGMT status, MMR status is not consistently determined in newly diagnosed GB tumors and therefore is not generally available to inform the treatment strategy. However, several studies have indicated that MMR deficiency—which potently protects tumor cells against TMZ irrespective of their MGMT status^11,12^—can emerge in response to treatment with the Stupp protocol and presents with some frequency in recurrent tumors.^14,15^ It is therefore relevant for clinical decision-making and would seem to exclude the further use of TMZ. In our MMR-deficient LN229TR2 model, NEO212 provided a much greater, strikingly synergistic benefit when added to RT. In fact, radiosensitization by NEO212 showed by far the greatest synergy in this particular model.

While the superior anticancer and radiosensitizing activity of NEO212 bodes well for its clinical application, an important question arose as to whether these striking activities perhaps came at the expense of greater toxicity. Our current results, in combination with earlier observations,^26–30^ seem to indicate otherwise. In several of our prior studies in mice, we showed that treatment with NEO212 in monotherapy fashion, even after extended cycles and elevated dosages, did not negatively impact the number of white blood cells (WBC), as determined by complete blood counts (CBC with differential), nor did it reveal any signs of liver or kidney damage. In a rat model, we applied greatly increased dosages of NEO212—in parallel to equal dosages of TMZ for comparison purposes—in an attempt to force the appearance of toxic signs.^30^ In these experiments, 100 mg/kg TMZ administered daily over 5 days caused a significant reduction in WBC counts, and increasing the dose to 200 mg/kg TMZ killed all 3 rats in this group. In comparison, none of the corresponding 2 treatment groups of rats administered with NEO212 revealed a significant reduction in WBC count, and all rats continued to thrive.^30^ When combined with radiation, NEO212 did not reveal any significant detrimental impact on the bone marrow or peripheral blood cell counts, further reinforcing our previous observations.

Several prior studies have confirmed that NEO212 maintains the alkylating function of its TMZ moiety.^24,26,40^ It was therefore surprising to find that this compound appeared to be better tolerated than TMZ yet exerted superior therapeutic benefit. While not yet entirely clarified, we surmise that at least part of NEO212’s superiority arises from its ability to better penetrate biological barriers, including the plasma membrane and the BBB. Experiments in mice confirmed what had been predicted from in silico analysis,^23^ namely that NEO212 is able to effectively cross the BBB; its brain:plasma ratio was found to be about 3-fold greater than that of TMZ^31^ and it exerted stronger anticancer activity in mouse models of GB and brain-metastatic breast cancer.^26–28^ Taken together, these studies propose a model where NEO212 is able to reach higher concentrations in the brain without concurrently causing increased exposure of the bone marrow. Because cell death resulting from DNA methylation caused by NEO212 (and TMZ) is dependent on cells undergoing active proliferation, one might reasonably expect that elevated drug concentrations in the brain would not cause neurotoxicity. At least in our mouse model, the addition of NEO212 to RT did not show signs of increased DNA damage in normal brain tissue. Nonetheless, this issue will require careful attention once NEO212 moves into the clinic.

Mechanistically, we expect the superior tumor accumulation of NEO212, when administered at the standard dosages, to translate into a more favorable on-target alkylation differential. This could conceivably overpower the repair capacity of the base-excision repair (BER) system in tumoral tissue, which, in turn, might allow for better synergisms to take place between unrepaired DNA methyl adducts and RT, irrespective of the MGMT presence or MMR status of the tumor. While TMZ preferentially kills via O6-MeG adducts,^6,19,41^ its ability to generate toxic n-alkylation events at standard dosages—which are primarily repaired by the BER system—is rather modest.^5,42^ Therefore, we expect the additional n-methylpurine adducts inflicted by NEO212 (eg, n7-methylguanine, n3-methyladenine, and n3-methylguanine) to be further exploited by RT and, thus, more efficiently, converted into lethal DSBs.^31^ However, rigorous comparative measurements of alkylation events by both NEO212 and TMZ, and specifically of the BER intermediates generated by these drugs during n-methylpurine alkylation events,^43^ are further warranted to prove this hypothesis. We believe an alkylation differential that favors NEO212 at standard dosages over TMZ is the most likely explanation for the observed differences in outcomes with these drugs in our animal models. Counterintuitively, NEO212’s requirement for RT to unfold its full DNA damaging effects also provides a plausible explanation for the absence of additional bone marrow toxicities observed with this drug.

Towards the clinical application of our promising findings, a Phase I trial is currently in progress for recurrent primary malignant gliomas and brain-metastatic cancer. Once a maximal tolerated dose is determined for Phase I, a Phase IIa study is planned for patients with recurrent, MGMT promoter unmethylated (ie, MGMT expressing) GB who have failed the Stupp protocol. A second Phase IIa study will be performed in newly diagnosed GB patients that are also MGMT unmethylated, where NEO212 will be combined with standard RT, along the principles of the Stupp protocol. It is expected that these 2 trials will provide answers as to whether NEO212 is clinically active in MGMT unmethylated tumors (both newly diagnosed and recurrent), and whether it is an effective, clinically useful radiation sensitizer.

## Supplementary Material

vdae095_suppl_Supplementary_Materials

## Data Availability

The original data from the study will be made available to all inquiring parties upon reasonable request. The data will be deposited in a public repository to which the authors will provide the means of access upon inquiry and whenever possible.
