# Supplementary material for: NEO212, temozolomide conjugated to NEO100, exerts superior therapeutic activity over temozolomide in preclinical chemoradiation models of glioblastoma
Source: Neurooncol Adv. 2024 Jun 11;6(1):vdae095. doi: 10.1093/noajnl/vdae095 (PMC11252566; doi:10.1093/noajnl/vdae095)

**NEO212, temozolomide conjugated to NEO100, exerts superior therapeutic activity over temozolomide in preclinical chemoradiation models of glioblastoma**

Radu O. Minea^1,4,^*, Thu Zan Thein^1^, Zhuoyue Yang^2^, Mihaela Campan^3^,

Pamela M. Ward^4^, Axel H. Schönthal^2^, and Thomas C. Chen^1,4,5,6^*

^1^ Department of Neurological Surgery, Keck School of Medicine (KSOM), University of Southern California (USC), Los Angeles, California (CA) 90089, USA.

^2^ Department of Molecular Microbiology and Immunology, KSOM, USC, Los Angeles, CA 90089, USA.

^3^ USC Clinical Laboratories, KSOM, USC, Los Angeles, CA 90089, USA.

^4^ Department of Pathology, KSOM, USC, Los Angeles, CA 90089, USA.

^5^ Norris Comprehensive Cancer Center, KSOM, USC, Los Angeles, CA 90089, USA.

^6^ NeOnc Technologies, Inc., Los Angeles, CA 90069, USA.

*** Corresponding authors:**

- Radu O. Minea, Department of Neurological Surgery, Keck School of Medicine, University of Southern California, 2011 Zonal Avenue, HMR 811, Los Angeles, California 90089, USA. Email: [minea@usc.edu](mailto:minea@usc.edu)
- Thomas C. Chen, Department of Neurological Surgery, Keck School of Medicine, University of Southern California, 1200 N. State St., Suite 3300, Los Angeles, California 90033, USA. Email: [Thomas.Chen@med.usc.edu](mailto:Thomas.Chen@med.usc.edu)

**ORCIDs**

- Radu O. Minea: 0000-0002-0929-6480
- Axel H. Schönthal: 0000-0003-0662-5653
- Thomas C. Chen: 0000-0002-2183-4667

**SUPPLEMENTAL MATERIALS AND METHODS**

**Reagents**

The anti-γH2AX antibody (clone JBW601) was purchased from EMD Millipore (Burlington, MA) and labeled with Alexa Fluor dye AF647 (ThermoFisher Scientific, Waltham, MA). A rat anti-mouse CD45 antibody (clone 30-F11) was purchased from Stem Cell Technologies (Cambridge, MA) and labeled with AF555 (ThermoFisher Scientific, Waltham, MA). An anti-GFP rabbit polyclonal antibody conjugated with AF488 and a mouse anti-human MGMT antibody (clone MT 23.2) were also purchased from Thermo Fisher Scientific, while the mouse-specific CD31 antibody (clone SZ31) was purchased from Dianova GmbH (Hamburg, Germany) and further labeled with AF647. A DeadEnd^TM^ fluorometric TUNEL kit was purchased from Promega (Madison, WI). The mouse anti-human MSH2 (MutS homolog 2, clone 3A2) and MSH6 (MutS homolog6, clone 3E1) antibodies were purchased from Cell Signaling (Danvers, MA).

**Cells**

USC02 MGMT knockdown cells (designated USC02 shMGMT) were prepared by stable infection with lentivirus construct purchased from VectorBuilder (Chicago, IL), which expresses a shRNA species that targets the 3’UTR region of human MGMT mRNA, along with a GFP reporter.

**Histological analyses of tissues**

Brain and bone marrow slides were imaged by confocal microscopy on an SP8 TCS Leica confocal microscope (Leica Microsystems, Deerfield, IL). High-content data was captured at various magnifications and further quantified digitally by pixel counting on images taken from multiple fields per tumor section using the HCImage software (Hamamatsu Corporation, Sewickley, PA).

**Analysis of hematologic toxicities**

Animals with USC02 xenografts were subjected to 10 days of treatment as described above. At the end of the treatment interval, bone marrow and blood were collected from all animals. Bone marrow was analyzed as described above; blood samples were submitted to Antech Diagnostics (Fountain Valley, CA) for white blood counts (WBC) and red blood counts (RBC).

**SUPPLEMENTAL FIGURE LEGENDS**

**Supplemental Figure 1. Tumor growth monitoring by optical imaging in the U251M model (MGMT-positive; lentivirus-expressed MGMT).** Animals implanted with GFP/Luc U251M cells were imaged at defined time intervals on an IVIS optical imaging system. The tumor burden is estimated qualitatively by measuring the photon flux intensity for each animal after inputting the average radiance (photons/s/cm^2^/sr) from the region of interest. Optical images generated at various time intervals post-tumor implantation are shown comparatively for all groups.

**Supplemental Figure 2. Tumor growth monitoring by optical imaging in the USC02 model (MGMT-positive; endogenous MGMT).** Animals implanted with GFP/Luc USC02 cells were imaged at defined time intervals on an IVIS optical imaging system. Photon flux intensity measurements were conducted as described above. Optical images generated at various time intervals post-tumor implantation are shown comparatively for all groups.

**Supplemental Figure 3. Tumor growth monitoring by optical imaging in the LN229TR2 model (MGMT-deficient, MMR-deficient).** Animals implanted with GFP/Luc LN229TR2 cells were imaged at defined time intervals on an IVIS optical imaging system. Photon flux intensity measurements were conducted as described above. Optical images generated at various time intervals post-tumor implantation are shown comparatively for all groups.

**Supplemental Figure 4. The generation of USC02 MGMT knockdown primary glioma stem cells.** (A) The map of the lentiviral construct used for MGMT knockdown shows the position of the short hairpin RNA species (inserted downstream of a U6 promoter) that was designed to target the 3’UTR of human MGMT mRNA. The lentiviral construct also expresses an enhanced GFP (EGFP) reporter gene, which was placed under a different promoter in a separate open reading frame (ORF). The latter was used to facilitate the sorting of infected cells by FACS. (B) USC02 cells infected with packaged lentiviral particles were FACS sorted twice based on GFP expression into a homogenous population. (C) The sorted USC02 shMGMT cells were further analyzed by Western blot for MGMT expression which confirmed the silencing of MGMT protein in this population.

**Supplemental Figure 5. Knocking down the MGMT protein in USC02 GB stem cells does not further sensitize this tumor model to the either NEO212 or TMZ treatments.** Kaplan–Meier survival plots were generated to display animal survival in response to various treatments. Groups of 5 mice were treated with NEO212 (25 mg/kg/day) or TMZ (25 mg/kg/day) alone or in combination with RT (2 Gy/day). Control groups of mice received vehicle only. Treatment started 14 days after implantation of tumor cells. All animals received the same treatment schedule for a total duration of 10 days (5-days on/2-days off/5-days on). Surprisingly, the survival data show that USC02 MGMT-knockdown tumors appear to respond to treatments similar to the wild-type tumors. The log-rank (Mantel-Cox) test was used for statistical comparisons. ns: not significant.

Supplemental Figure 1


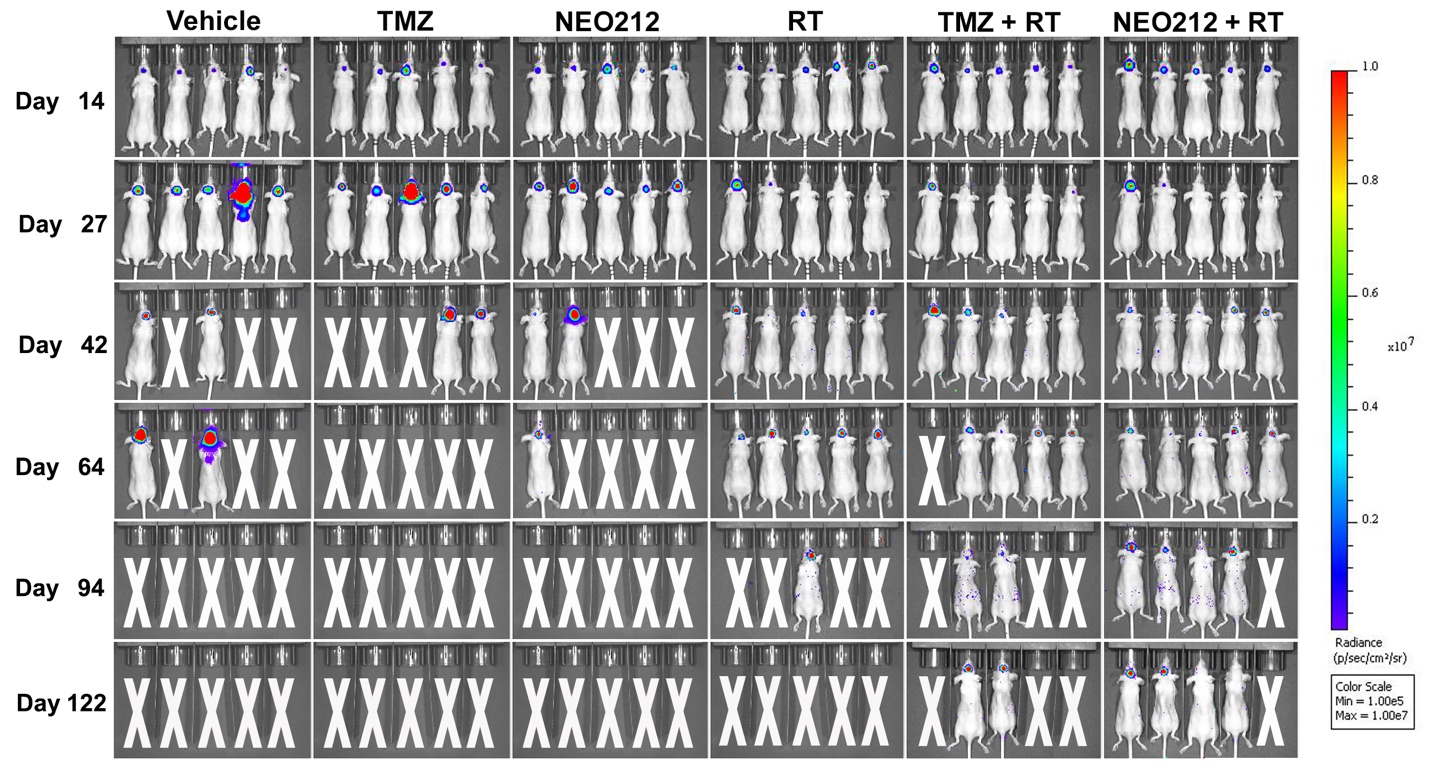


Supplemental Figure 2


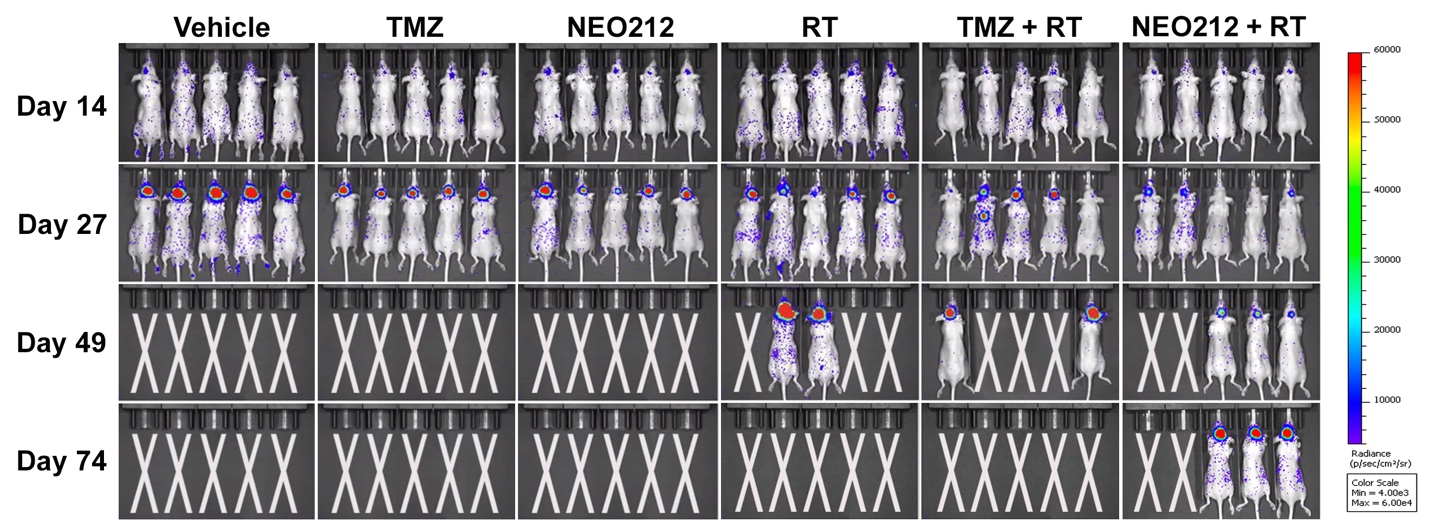


Supplemental Figure 3


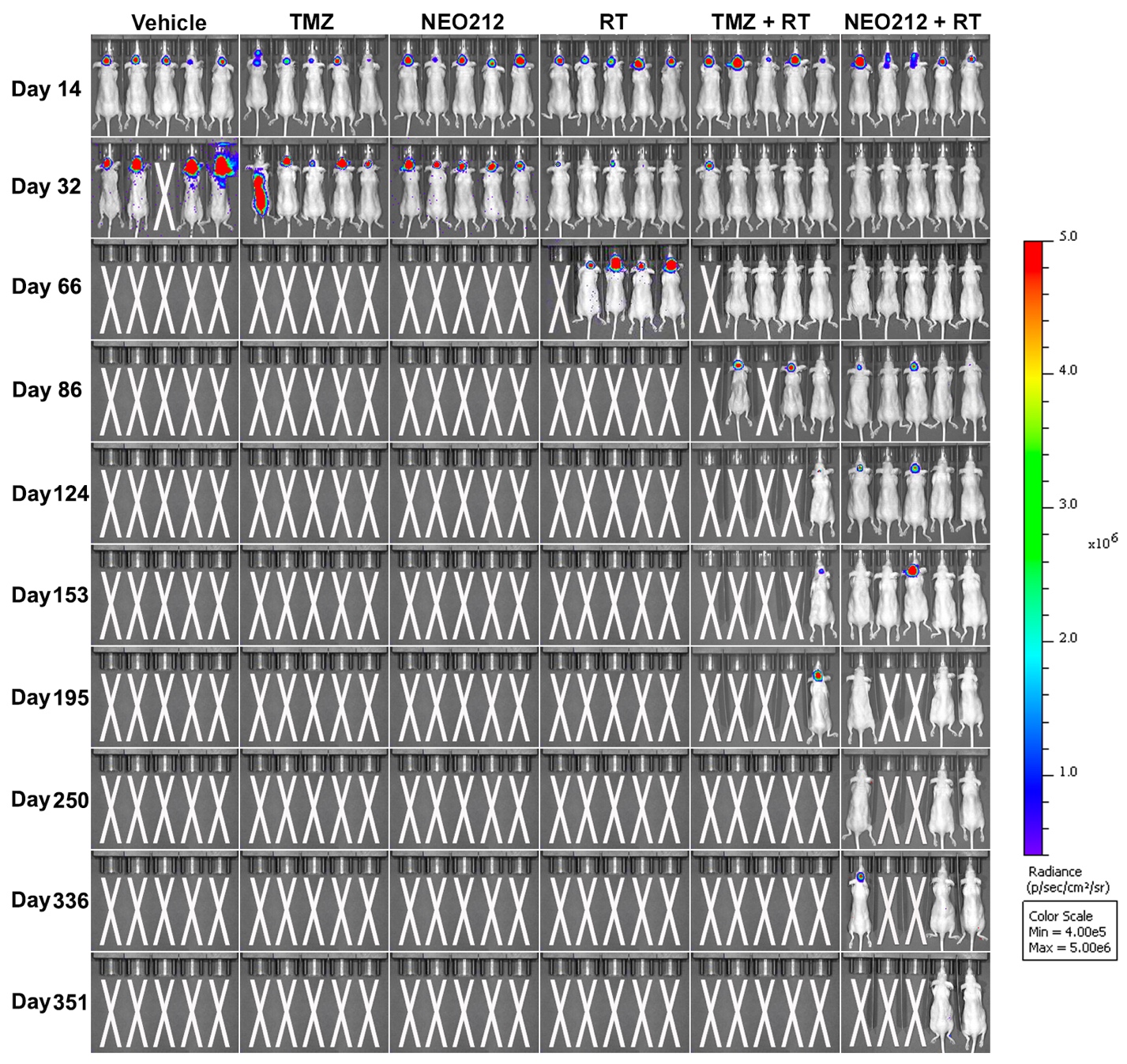


Supplemental Figure 4


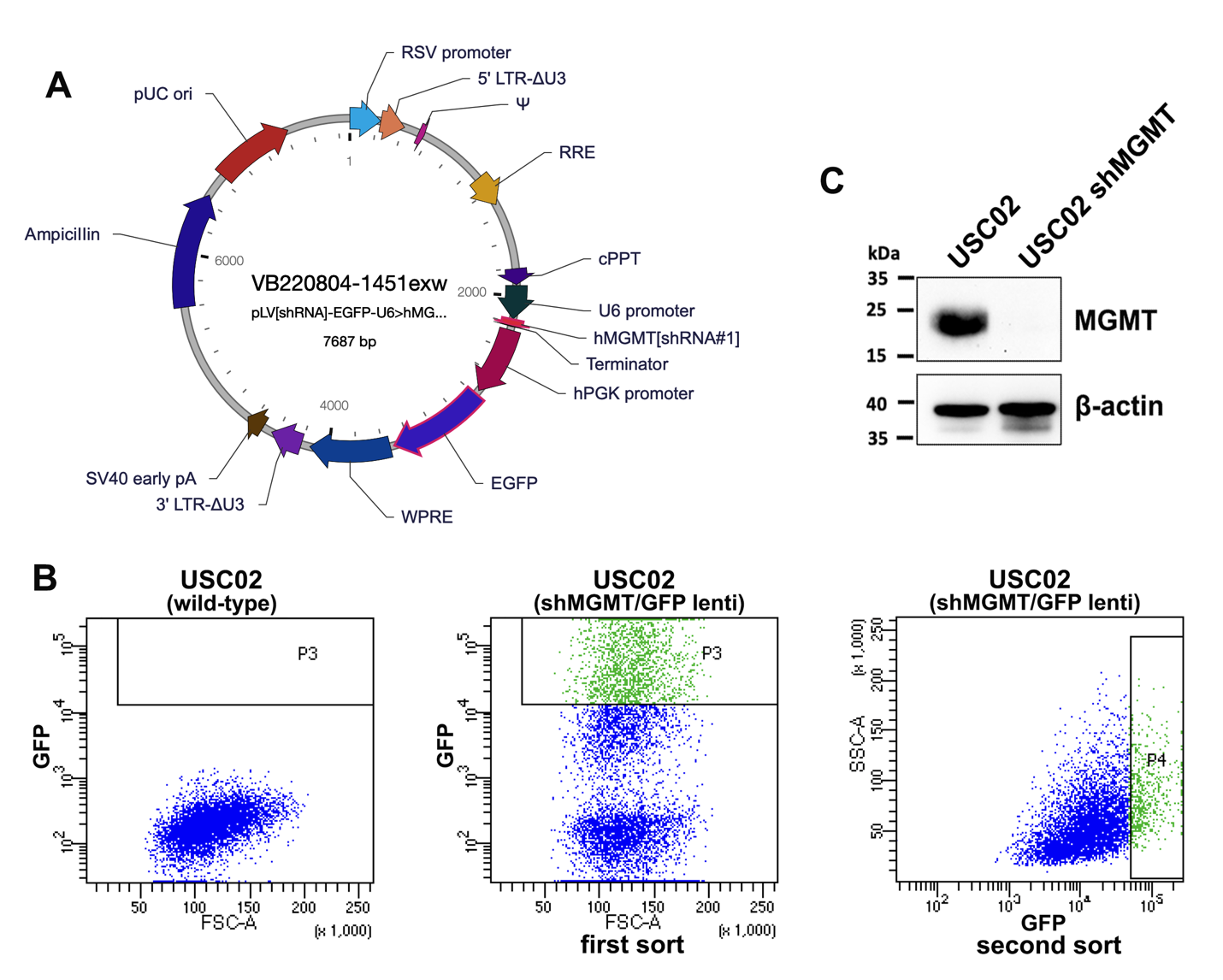


Supplemental Figure 5


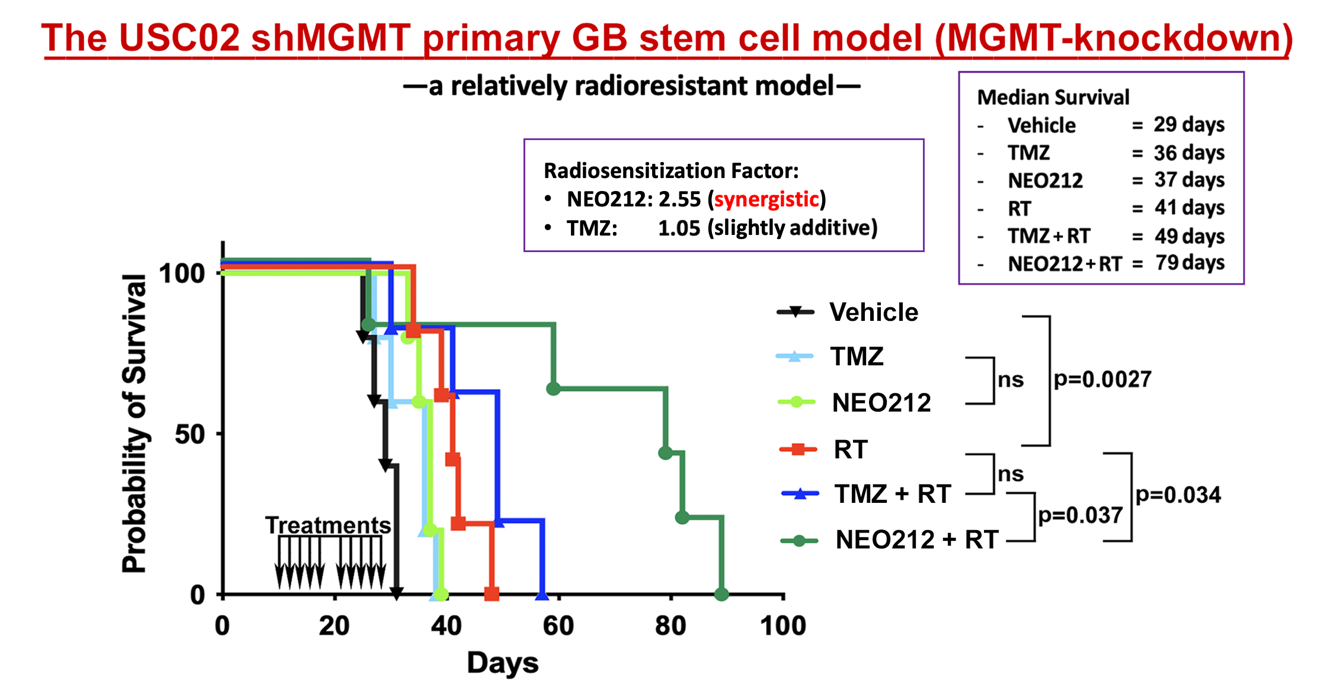

Supplement: vdae095_suppl_Supplementary_Materials [file vdae095_suppl_supplementary_materials.docx]
